# In Vitro Proliferation of Adult Human Beta-Cells

**DOI:** 10.1371/journal.pone.0035801

**Published:** 2012-04-26

**Authors:** Sabine Rutti, Nadine S. Sauter, Karim Bouzakri, Richard Prazak, Philippe A. Halban, Marc Y. Donath

**Affiliations:** 1 Department of Genetic Medicine and Development, University of Geneva, Geneva, Switzerland; 2 Department of Biomedicine and Division of Endocrinology, Diabetes and Metabolism, University Hospital Basel, Basel, Switzerland; University of Bremen, Germany

## Abstract

A decrease in functional beta-cell mass is a key feature of type 2 diabetes. Glucagon-like peptide 1 (GLP-1) analogues induce proliferation of rodent beta-cells. However, the proliferative capacity of human beta-cells and its modulation by GLP-1 analogues remain to be fully investigated. We therefore sought to quantify adult human beta-cell proliferation *in vitro* and whether this is affected by the GLP-1 analogue liraglutide.

Human islets from 7 adult cadaveric organ donors were dispersed into single cells. Beta-cells were purified by FACS. Non-sorted cells and the beta-cell enriched (“beta-cells”) population were plated on extracellular matrix from rat (804G) and human bladder carcinoma cells (HTB9) or bovine corneal endothelial ECM (BCEC). Cells were maintained in culture+/−liraglutide for 4 days in the presence of BrdU.

Rare human beta-cell proliferation could be observed either in the purified beta-cell population (0.051±0.020%; 22 beta-cells proliferating out of 84'283 beta-cells counted) or in the non-sorted cell population (0.055±0.011%; 104 proliferating beta-cells out of 232'826 beta-cells counted), independently of the matrix or the culture conditions. Liraglutide increased human beta-cell proliferation on BCEC in the non-sorted cell population (0.082±0.034% proliferating beta-cells vs. 0.017±0.008% in control, p<0.05).

These results indicate that adult human beta-cell proliferation can occur *in vitro* but remains an extremely rare event with these donors and particular culture conditions. Liraglutide increases beta-cell proliferation only in the non-sorted cell population and only on BCEC. However, it cannot be excluded that human beta-cells may proliferate to a greater extent in situ in response to natural stimuli.

## Introduction

Type 2 diabetes is characterized by a progressive decrease in functional beta-cell mass that can no longer compensate for insulin resistance [1], [2]. In this context, the ability of murine and rodent beta-cells to proliferate as one possible route to compensate for the decrease in beta-cell mass has been extensively studied [3]. However, whether adult human beta-cells are able to proliferate remains under debate [4]–[7]. A low rate of human beta-cell proliferation has been observed in vivo [8]–[10] in pancreatic sections. In vitro, some studies failed to detect any adult human beta-cell proliferation [4], whereas others observed proliferation at a rate >1% [6]. The ability of beta-cells to proliferate in vitro varies depending on their contact to different extracellular matrices [4], [11], [12] which might explain some of the discrepancies between studies from various research groups.

Liraglutide is a long-acting glucagon-like peptide-1 (GLP-1) derivative that has been shown to preserve beta-cell mass by inducing proliferation of rat and mouse beta-cells [4], [8]. However, the proliferative capacity of human beta-cells and its modulation by GLP-1 analogues remains to be fully investigated.

The aim of this study was to determine whether human beta-cells are able to proliferate *in vitro* under different culture conditions and whether this may be modulated by the GLP-1 analogue liraglutide. To answer these questions, sorted human beta-cells and human non-sorted islet cells were cultured on 3 different matrices, each with 4 different treatment conditions and in the continuous presence of BrdU. These experiments were conducted by two independent research groups under the same conditions regarding the timeline of the experiments, the origin of the matrices and the reagents used as well as the method with which the cells were counted.

## Methods

### Human islet isolation and culture

**Figure 1:**
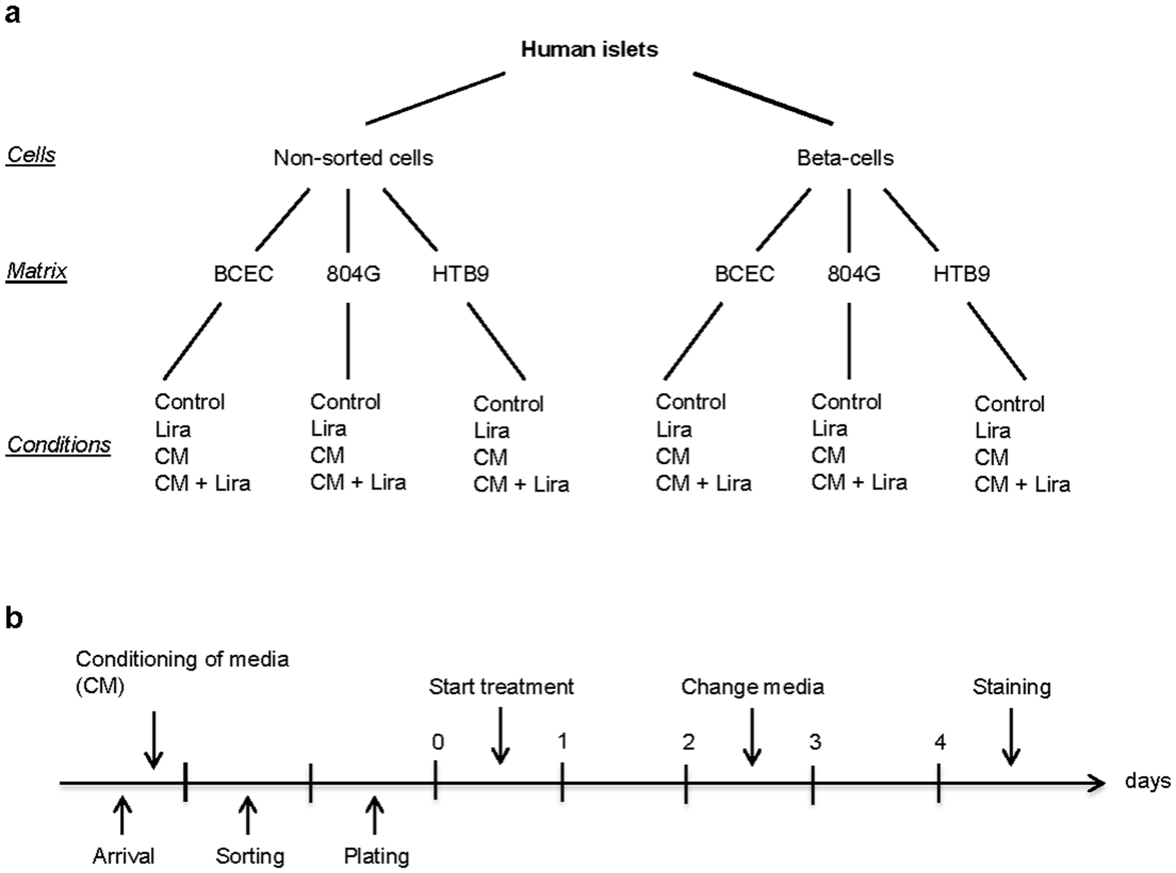
Overview and timeline of the experiments. Overview (a) and timeline (b) of the experiments showing the different cell fractions, matrices and treatments evaluated. CM, conditioned medium; Lira, Liraglutide; BCEC, bovine corneal endothelial cell; 804G, rat bladder carcinoma; HTB9 human bladder carcinoma.

Human islets were isolated from pancreata of organ donors at the University of Geneva Medical Centre (Switzerland) and at the University of Milan (Italy) (n = 7; Age average: 49.3 years; (range 18–66); BMI average: 26.5 (range 21.6–36.9)). Human islets were provided by the islet for research distribution program through the European Consortium for Islet Transplantation, under the supervision of the Juvenile Diabetes Research Foundation (31-2008-416) Islets were obtained in Milano with patient consent governed by the laws of the Nord Italia Transplant Program, with ethical approval from the Comitato Etico Istituto Scientifico San Raffaele. Islets were obtained in Geneva with ethical approval from the N.A.C. (Neuclid, Apsic (Anesthesiology, Pharmacology, Surgery intensive care), Surgery) Joint Departmental Ethical Committee of the University Hospital and according to Swiss legislation on cadaveric tissue procurement. Human islets used in this study were analyzed anonymously. Human islets were cultured in CMRL-1066 medium containing 5 mmol/l glucose, 100 units/ml penicillin, 100 µg/ml streptomycin, 2 mM glutamax, 250 µg/ml gentamycine, 10 mM Hepes and 10% FCS (Invitrogen, Basel, Switzerland). The islets were cultured overnight in suspension dishes at a concentration of 500 islets equivalent/ml and collected by centrifugation; the supernatant was kept as ‘conditioned medium’ at 4°C. To obtain single cells, islets were dispersed with Accutase (PAA Laboratories, Pasching, Austria) for 10 minutes at 37°C. Beta-cells were purified using a FACSVantage (Becton Dickinson, Sunnyvale, CA, USA) as described [4] by virtue of their zinc content using the zinc-binding fluorochrome Newport Green. Non-sorted single cells and sorted beta-cells were cultured on extracellular matrix-coated plates derived from bovine corneal endothelial cells (BCEC, Novamed, Jerusalem, Israel), 804G or HTB9 as described elsewhere [4] and were left in islet media for 24 h to adhere and spread before initiation of the experiments. Cells were treated with Liraglutide (100 nM, kindly provided by Novo Nordisk, Denmark) and/or conditioned media. Medium was changed after 2 days of culture (Fig. 1).

### Proliferation of islet cells

BrdU was added to the cells at the start of the treatment. After 4 days treatment, the incorporated BrdU was detected with an anti-BrdU antibody (dilution 1/10, incubation 30 minutes at 37°C, Roche, Basel, Switzerland). The single cells were co-stained with a guinea pig anti-insulin antibody (Dako, Glostrup, Denmark, Dilution 1/50, 30 minutes at 37°C) and DAPI to stain all nuclei. For each condition in each individual experiment, the total cell number, insulin-positive cells, BrdU-positive cells and double positive insulin/BrdU cells (considered as proliferating beta-cells) present on each dish were counted blindly with Image J (NIH, Bethesda, Maryland, USA) and Adobe Photoshop CS4 (Adobe Systems, San Jose, California, USA) on pictures taken with fluorescent microscopes (AxioZ1, Zeiss, Jena, or Olympus BX61, Hamburg, Germany), in parallel by 2 different investigators in Geneva and Basel. Each n represents a different human islet donor.

### Statistical analysis

Data are expressed as means ± SEM. All data were tested for normality and analyzed with PRISM (GraphPad, San Diego, CA). Significance was tested using Student's *t* test and Mann-Whitney for multiple comparison analysis. Significance was set as *P*<0.05.

## Results

Non-sorted dispersed human islet cells and sorted human beta-cells were cultured on 3 different extracellular matrix-coated dishes in the presence of BrdU and treated with Liraglutide and/or conditioned medium. After 4 days of treatment, cells were co-immunostained for BrdU incorporation and insulin. The number of total cells present on the dishes at the end of the experiment was not changed by the matrices or any of the treatments as determined by DAPI staining. Similarly, the percentage of proliferating insulin-negative cells observed on dishes containing non-sorted cells (0.164±0.029%) was independent of matrices and treatments. In the sorted beta-cell fraction, a purity of 98.63±0.19% was obtained at the end of the culture period, and this too was unaffected by the different matrices or treatments.

### Proliferation of sorted beta-cells is not influenced by matrix or treatment

Neither Liraglutide nor conditioned medium nor the combination of the 2 treatments had any effect on the percentage of BrdU/insulin-double positive cells (data not shown). There were also no significant differences in beta-cell proliferation among the 3 different matrices (Table 1). This allowed for the data from all conditions to be pooled, with a total of 22 proliferating beta-cells out of 84,283 beta-cells scored in 7 independent experiments, resulting in an overall rate of 0.051±0.020% (Table 1).

**Table 1:** Beta-cell proliferation and number of total cells evaluated in sorted human beta-cells and non-sorted dispersed islet cells cultured on 3 different matrices in control medium or with Liraglutide and/or conditioned medium (n = 7).

		Sorted beta-cells	Non-sorted cells			
		Pooled treatments^a^	Control	Lira	CM	CM+Lira
% BrdU^+^/Ins^+^	BCEC	0.034±0.019	0.017±0.008	0.082±0.034 *	0.042±0.014	0.027±0.014
	804 G	0.015±0.004	0.032±0.026	0.011±0.005	0.049±0.029	0.186±0.088
	HTB9	0.097±0.082	0.028±0.025	0.047±0.034	0.097±0.040	0.035±0.024
		Sorted beta-cells	Non-sorted cells			
Number of cells counted	Total cells	85'412	525'001			
	Beta cells	84'283	232'826			
	BrdU^+^/Ins^+^	22	104			
% BrdU^+^/Ins^+^	Pooled matrices and treatments	0.051±0.020	0.055±0.011			

a For each matrix, the numbers of proliferating beta-cells were pooled from dishes treated with control media ±Liraglutide or condit.media ± Liraglutide.

* represents p<0.05 as tested by Mann-Whitney.

CM, conditioned medium; Lira, Liraglutide; BCEC, bovine corneal endothelial cell; 804 G, rat bladder carcinoma; HTB9, human bladder carcinoma.

### Liraglutide induces beta-cell proliferation only in non-sorted cells on BCEC

The non-sorted single cells contained 52.37±2.03% beta-cells, which was not changed by any of the various conditions. We observed a total of 104 proliferating beta-cells out of 232'826 beta-cells or 0.055±0.011%, similar to that found in sorted beta-cells (Table 1, last row). Liraglutide alone increased BrdU incorporation only in cells cultured on BCEC (0.017±0.008 vs 0.082±0.034 control vs Liraglutide, p<0.05). On this matrix, cells from 6 out of 7 donors showed an increase in beta-cell proliferation in response to Liraglutide. There was no significant difference among any of the other conditions (treatment or matrices).

Taken together, we observed rare *in vitro* proliferating human beta-cells in sorted cells as well as in dispersed islets. The rate of proliferation was unchanged by different treatment and matrices. Liraglutide increased beta-cell proliferation only in non-sorted cells and only on BCEC.

## Discussion

In this study we found that adult human beta-cells proliferate *in vitro*. However, this remains an extremely rare event. We used BrdU incorporation which allowed us to detect the proliferation that had arisen over 4 days, whereas the other staining methods for proliferation only detect the cells replicating at the time of fixation. Given the rarity of this event and despite the very large numbers of cells observed, the use of any other staining method than BrdU would likely have precluded our ability to quantify proliferation. However, BrdU does not discriminate between proliferating cells and incorporation due to repair following DNA damage, indicating that our data regarding human beta-cell proliferation may if anything be an over-estimate.

Our results are in line with results from others [5]–[7] but clearly diverge from our earlier study [4]. In these experiments, proliferating human beta-cells could not be detected at all, also not in the presence of different growth factors and hormones including Liraglutide. This discrepancy may be explained by the rarity of the event in combination with a different microscopy and counting method, as well as the smaller number of cells examined for each donor.

The proliferation observed in our study was not influenced by islet conditioned medium or the presence of other islet cells. Our data thus showed that purified human beta-cells (purity >98%) are able to proliferate *in vitro* to the same extent as beta-cells surrounded by the various other islet cell types. Our results did not tell us the origin of the proliferating beta-cells. However the similar proliferation rate between beta-cells in the sorted and in the non-sorted fractions suggests that self-replication or dedifferentiation-proliferation-redifferentiation could be more probable routes for proliferation than neogenesis from precursor cells or transdifferentiation.

These data with rare proliferative adult human beta-cells do not allow us to draw any conclusions regarding beta-cell proliferation *in vivo* in adult humans, and the potential beneficial effect of GLP-1-based therapy in such a context. Moreover, it cannot be excluded that human beta-cells may proliferate to a greater extent in situ in response to natural physical and humoral stimuli within the physiological setting of the islet. In this series of 7 donors, the majority were above 50 years old, only 2 donors were younger (18 and 38 respectively). This series did not allow us to observe any correlation or trend between proliferation and age. Previous studies have shown a decrease in adult human beta-cell proliferation with age ([6], [8]–[10]. These correlations have been observed on pancreatic sections where tissues from very young (<10 years) or very old (>80 years) patients were included; these are age groups from which isolated islets are typically not available. Our study also differs in methodology from a previous study [6] where age-dependent effects have been observed on cultures of whole islets.

In the present study, the baseline proliferation of adult human beta-cells was comparable among the 3 different matrices used. These extracellular matrices may not offer the optimum substratum for human beta-cell proliferation and are in any case not similar in composition or structure to the naturally occurring basement membrane surrounding human beta-cells [4], [11], [13]. Interestingly, Liraglutide increased proliferation of adult human beta-cells only in the non-sorted cell fraction cultured on BCEC.However, even in the presence of this GLP-1 analogue and BrdU throughout the 4 day culture period, <0.1% of beta-cells were seen to have proliferated. The results obtained here with human adult beta-cells differ clearly from those obtained previously with rat or mouse beta-cells [4], [14]. There, replication is considered to be the main mechanisms for the maintenance of beta-cell mass with rates of proliferation predicted to reach >20% under these same conditions in response to GLP-1. There thus is a striking difference between rodent and adult human beta-cells in their capability to proliferate. This raises the question of the relevance of mouse or rat beta-cells as a model for studying the impact of pharmacological compounds on human beta-cell proliferation.
